# Effects of polyphenols extracted from Keemun black tea on CYP450s activity and molecular mechanisms

**DOI:** 10.1002/fsn3.4319

**Published:** 2024-07-19

**Authors:** Dan Zuo, Le Lv, Hong Ren, Haiyan Sun

**Affiliations:** ^1^ School of Food and Drug Shenzhen Polytechnic University Shenzhen Guangdong China; ^2^ Vocational and Technical College of Anshun Anshun Guizhou China; ^3^ School of Applied Biology Shenzhen Institute of Technology Shenzhen Guangdong China

**Keywords:** CYP450, food–drug interaction, Keemun black tea, polyphenol PXR

## Abstract

Keemun black tea (KBT) is a luxurious traditional tea in China that has been commonly consumed because of its superior aroma and special taste. However, the risks remain unknown when KBT is used concomitantly with other drugs or food products. Therefore, we aimed to explore the effect of the tea polyphenols from KBT on the protein and mRNA levels of CYP450 and related mechanisms. The extraction of tea polyphenols from KBT and the content and component analysis of polyphenols were performed. A total of 24 female C57BL/6J mice were given tea polyphenols (0, 75, 150, 300 mg/kg) for 7 days, respectively. Liver tissues were collected 2 h after the last administration. The expression of Cyp3a11, Cyp1a2, Cyp2e1, Cyp2c37, and PXR mRNA was detected by real‐time PCR, and the expression of Cyp3a11, Cyp1a2, Cyp2e1, Cyp2c37, and PXR protein was detected by Western blotting. A transient co‐transfection reporter gene assay on HepG2 cells was also used to verify the role of PXR in regulating CYP3A4 expression. Our results showed that tea polyphenols from KBT significantly induced the expression of CYP 3A11 and PXR in general, inhibited the expression of Cyp1a2 and Cyp2e1 in general, and significantly inhibited the mRNA expression of Cyp2c37 but induced its protein expression. The reporter gene‐transfected cells demonstrated that tea polyphenols could enhance the PXR‐mediated transactivation of the CYP3A4 promoter via rifampicin‐induction. Meanwhile, tea polyphenols could significantly accelerate CYP3A11/3A4 expression by activating the PXR‐CYP3A4 pathway. In conclusion, KBT polyphenols could significantly affect the expression of various subtypes of the Cyp450 enzyme in mice livers via the PXR‐CYP450 pathway, suggesting that metabolism‐based interactions can occur when they are used in combination with medicines.

## Graphical abstract

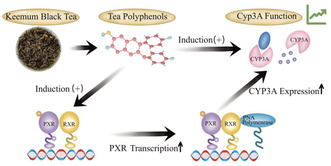

## 1 INTRODUCTION

Keemun black tea (KBT) is a luxurious traditional tea in China that has been commonly consumed because of its superior aroma and special taste (Guo et al., 2018). It contains more than 30 types of tea polyphenols and accounts for 15%–30% of the dry weight (DW) of tea leaves. These polyphenols play a leading role in the main biological and pharmacological activities of tea (Stodt & Engelhardt, 2013; Zhang, Santos, et al., 2019). Consumption of KBT has increased dramatically over the last few decades, and there is a need to understand the interactions between tea and drugs.

Accumulative evidence has shown that tea‐drug interactions may be induced by two major mechanisms: exogenous active substances directly affect (i) the functional activities of drug metabolism enzymes and/or transporters and (ii) the transcriptional levels of xenobiotic metabolizing enzymes. Cytochromes P450 (CYPs) are a superfamily of enzymes that are responsible for phase I drug metabolism (Kamble et al., 2020). Four isoforms, Cyp3a11, Cyp1a2, Cyp2c37, and Cyp2e1, are involved in the metabolism of more than 90% of drugs in the clinic, and they can also be regulated by several xenobiotics (Gerbal‐Chaloin et al., 2006). Mouse and human drug metabolizing enzyme genes are homologous, that is, mouse liver Cyp3a11, Cyp1a2, Cyp2c37, and Cyp2e1 are equivalent to human Cyp3A4, Cyp1A2, Cyp2C9, and Cyp2E1 (Hrycay & Bandiera, 2009). Pregnane X receptor (PXR) is a pivotal transcriptional modulator of Cyp3A11 and CYP3A4, which underlies the pharmacokinetics of food–drug interaction (Bulutoglu et al., 2019; Mackowiak et al., 2018; Mani et al., 2013). It has been reported that PXR can regulate both exogenous and drug‐induced expression levels of CYP2B, CYP2C, and CYP3A subfamilies, and CYP3A4 is the most well‐characterized one among the CYP3A family (Manda et al., 2017).

The consumption of nutritional supplements based on tea extracts has increased dramatically in recent years, largely due to their health benefits (e.g., antioxidants). However, there are unknown risks when KBT is used concomitantly with other drugs or food products (Feltrin & Oliveira Simões, 2019; Satoh et al., 2016). At present, the previous research on tea polyphenols mainly focuses on their biological activities, such as antioxidant, anti‐tumor, and bacteriostasis (Dekant et al., 2017). However, the effects of KBT on drug pharmacokinetic profiles and metabolizing enzymes have yet to be described. Only a few reports have been found on green tea extracts and their catechin compounds (Albassam & Markowitz, 2017). Based on different experimental conditions, the modulatory effects of tea polyphenols on the CYP450 enzyme have been inconsistent or contradictory, which may be attributed to different experimental designs, administration, routes of administration, and dosage forms (Yang & Pan, 2012). Hence, we aimed to explore the potential effects of tea polyphenolic extract from KBT on CYP450 enzyme activities and determine the possible role of PXR. The extraction and component analysis of tea polyphenols from KBT were performed. Mice were given tea polyphenols, and liver tissues were collected for qRT‐PCR and Western blotting. A transient co‐transfection reporter gene assay on HepG2 cells was also performed.

## 2 MATERIALS AND METHODS

### 2.1 Chemicals and reagents

A tea factory in Qimen County (Anhui, China) provided the KBT samples. HepG2 cells were procured from ATCC (MD, USA). The pSG5‐hPXR expression vector and pGL3‐CYP3A4‐XREM luciferase reporter were provided generously by Dr. Steven Kliewer (University of Texas Southwestern Medical Center, TX, USA) and Dr. Jeff Staudinger (Department of Pharmacology and Toxicology, University of Kansas, KS, USA), respectively. The pRL‐TK plasmid, pSG5 empty vector, and *Escherichia coli* DH5a strain were supplied by Beijing Tianenze Gene Technology Co., Ltd. (Beijing, China). Rifampicin (RIF), dimethyl sulfoxide (DMSO), 100× protease inhibitor cocktail, acetonitrile, folic‐phenol reagent, gallic acid, epicatechin, epigallocatechin, catechin, and catechin gallate were collected from Sigma‐Aldrich (MO, USA). DMEM and FBS were obtained from Gibco (Grand Island, NY, USA) and Gemini (Grand Island, NY, USA). All other reagents were obtained from Sigma (St. Louis, MO, USA) unless otherwise noted.

### 2.2 Extraction of tea polyphenols

The tea leaves were dried in an oven at 80°C until a constant weight was achieved and then crushed into a fine powder of approximately 80 mesh and kept in a glass container before analysis. The KBT powder was mixed with 70% methanol at 1:25 (m/V) and subjected to condensation reflux extraction for 30 min (repeated extraction 3 times). After filtration, rotation, and evaporation to 15.2% of the original volume, the sample was again extracted with 3 times the volume of chloroform and then rotary evaporated. The extract was evaporated to about 5% of the volume after extraction, and the rest was dissolved in ultrapure water. After centrifugation, the supernatant was dried and stored in a desiccator.

### 2.3 Determination of total polyphenol extract

The total polyphenol content of KBT extract was evaluated by the Folin–Ciocalteu method with slight modifications (Minussi et al., 2003). Briefly, 30 μL of the sample, 100 μL of Folin–Ciocalteu, 1 mL of sodium carbonate (10%), and 800 μL of distilled water were mixed and then incubated at 37°C for 1 h. The absorbance was recorded at 760 nm. The calibration curve was constructed with gallic acid solution, and the data are presented as mg of DAE per gram of DW.

### 2.4 Detection of the main components of KBT polyphenols

The major components of KBT polyphenols were detected by an LC‐20A Shimadzu HPLC system (Shimadzu Technologies, Kyoto, Japan) consisting of an autosampler, diode array detector, integrated vacuum degasser, Infinity binary pump, and thermostated column compartment. The Agilent ZORBAX Eclipse Plus Phenyl‐Hexyl (5 μm, 4.6 mm × 250 mm; Agilent Technologies, CA, USA) was employed as an analytical column. The mobile phase contained 0.17% (v/v) acetic acid–water (A) and acetonitrile (B). The detection wavelength and flow rate were 280 nm and 1.0 mL/min, respectively. The following gradient elution was applied: 0–10 min, 2%–3% B; 10–15 min, 3%–7% B; 15–30 min, 7%–10% B; 30–55 min, 10%–12% B; 55–65 min, 12%–14% B; 65–75 min, 14%–15% B, and 75–80 min, 15%–25% B. The column temperature remained constant at 35°C, and the injection volume was 10 μL per sample.

### 2.5 Animals

Female C57BL/6J mice, six weeks old, were supplied by the Experimental Animal Center of Southern Medical University (Guangdong, China, Animal Certificate No: SCXK (Guangdong) 2011‐0015). All mice were given unlimited access to standard rodent chow and clean tap water, reared under a 12/12‐h dark/light cycle at 25 ± 0.2°C and 55%–60% relative humidity. After 7 days of acclimatization feeding, 24 mice were randomly divided into 4 groups (*n* = 6). In the control group, the mice were orally administered with water alone. Those in the KBT polyphenol groups were orally administered with different concentrations of KBT polyphenols (75, 150, and 300 mg/kg). On the 7th day, all mice were sacrificed 2 h after the final administration. Liver tissues were harvested and stored at −80°C until use. All experiments were approved by the Ethics Committee of Shenzhen Polytechnic according to the Regulations of Experimental Animal Administration issued by the Ministry of Science and Technology, China (http://www.most.gov.cn).

### 2.6 RNA isolation and reverse transcriptase real‐time PCR

TRIzol reagent (Invitrogen, CA, USA) was utilized to isolate the total RNA by following the manufacturer's instructions. The RNA sample concentration was determined using UV absorbance spectrophotometry (Shandong Huanmei Analytical Instrument Co., Ltd., Weifang, China) at 260 nm, while their integrity and purity were assessed by ethidium bromide‐stained agarose‐formaldehyde gel electrophoresis. The RNA samples (2 μg) were then reverse‐transcribed using a High‐Capacity cDNA Archive Kit (Takara). Real‐time PCR was conducted on a Light Cycler 2.0 Real‐Time Detection System (Roche, CA, USA) using the SYBR®Premix Ex Taq™ kit (Takara). The cycling conditions were as follows: 95°C for 30 s, followed by 40 cycles of 95°C for 3 s and 60°C for 30 s. All primers were commercially synthesized by Sangon Biotech (Shanghai, China), and their sequences are listed in Table 1. The 2^(‐ΔΔCt)^ method was used to calculate the mRNA expression levels of *Cyp450* and *PXR* after normalizing with those of *β‐actin*.

**TABLE 1:** Primer sequences used for real‐time polymerase chain reaction.

Genes	Primer sequence (5′‐3′)		Product size (bp)	Annealing (°C)	Cycle
Cyp3a11	Forward primer	ACAAACAAGCAGGGATGGAC	84	55.5	40
	Reverse primer	GGTAGAGGAGCACCAAGCTG		58.4	
Cyp2c37	Forward primer	GTGGCCAGGGTCAAATTTCTC	51	58.4	40
	Reverse primer	CTGCATGACAGCACGGAGTT		56.7	
Cyp1a2	Forward primer	TGGAGCTGGCTTTGACACAG	71	58.2	40
	Reverse primer	CGTTAGGCCATGTCACAAGTAGC		58.4	
Cyp2e1	Forward primer	CCTGCTGCCCATCATTATCC	84	56.2	40
	Reverse primer	GCTCTTACCCACTGAGCCATCT		59.2	
PXR	Forward primer	GTTCAAGGGCGTCATCAACT	122	55.4	40
	Reverse primer	TCGTGTTGAACCTCAGGATG		54.6	
β‐Actin	Forward primer	TGTTACCAACTGGGACGACA	165	56.0	40
	Reverse primer	GGGGTGTTGAAGGTCTCAAA		54.9	

### 2.7 Protein isolation and Western blotting

The liver tissues were collected and washed with ice‐cold RIPA buffer (pH 7.4) with 1.0 mM EDTA, 0.125 M potassium chloride, and a protease inhibitor. After centrifugation at 1000 r/min for 10 min, the supernatant was obtained and detected by the BCA protein assay kit (Pierce, Rockford, IL, UAS). Protein was separated on a 10% SDS‐PAGE and then transferred electrophoretically onto a PVDF membrane. The membrane was probed with anti‐Cyp3a11, anti‐β‐actin, anti‐Cyp2c37, anti‐Cyp1a2, anti‐Cyp2e1, or anti‐PXR antibodies (Abcam, Cambridge, MA, USA). Chemiluminescent detection was performed on an X‐ray film using an enhanced chemiluminescence substrate (Pierce Chemical, IL, USA). Image Lab software version 5.1 was used to quantitatively analyze (densitometry) the stained protein bands. This experiment was repeated at least three times, and a representative Western blot was done.

### 2.8 Transient transfection and reporter gene assay

Transiently transfected HepG2 cells were employed to evaluate the transcriptional activity of PXR. HepG2 cells were cultured in DMEM with 10% FBS. The reporter plasmid was transiently transfected into HepG2 cells by using Lipofectamine® 2000 transfection reagent (Invitrogen, CA, USA). Briefly, the cells were seeded in 96‐well plates at a concentration of 1 × 10^4^ cells per well, followed by stabilization for 24 h. Then, the cells were transfected with 100 ng of pSG5‐hPXR or empty vector, 100 ng of the CYP3A4‐XREM luciferase reporter gene, or 6 ng of pRL‐TK (internal standard). Following 24 h of transfection, the cells were treated with different concentrations (100, 200, and 300 μg/mL) of KBT polyphenols or 0.1% DMSO for another 24 h. The Dual‐Luciferase Reporter Assay System (Promega, WI, USA) was utilized to measure the luciferase activities. The values were presented as fold induction relative to the activities in hPXR‐transfected cells.

### 2.9 Statistical analysis

All statistical analyses were conducted using SPSS 26.0 software (SPSS Inc., IL, USA). The data were expressed as the mean ± standard deviation (SD). The difference between experimental and control groups was compared by an unpaired Student's *t*‐test or one‐way ANOVA followed by Dunnett's test. The level of statistical significance was considered at *p* < .05.

## 3 RESULTS

### 3.1 Content and composition analysis of KBT polyphenols

**FIGURE 1:**
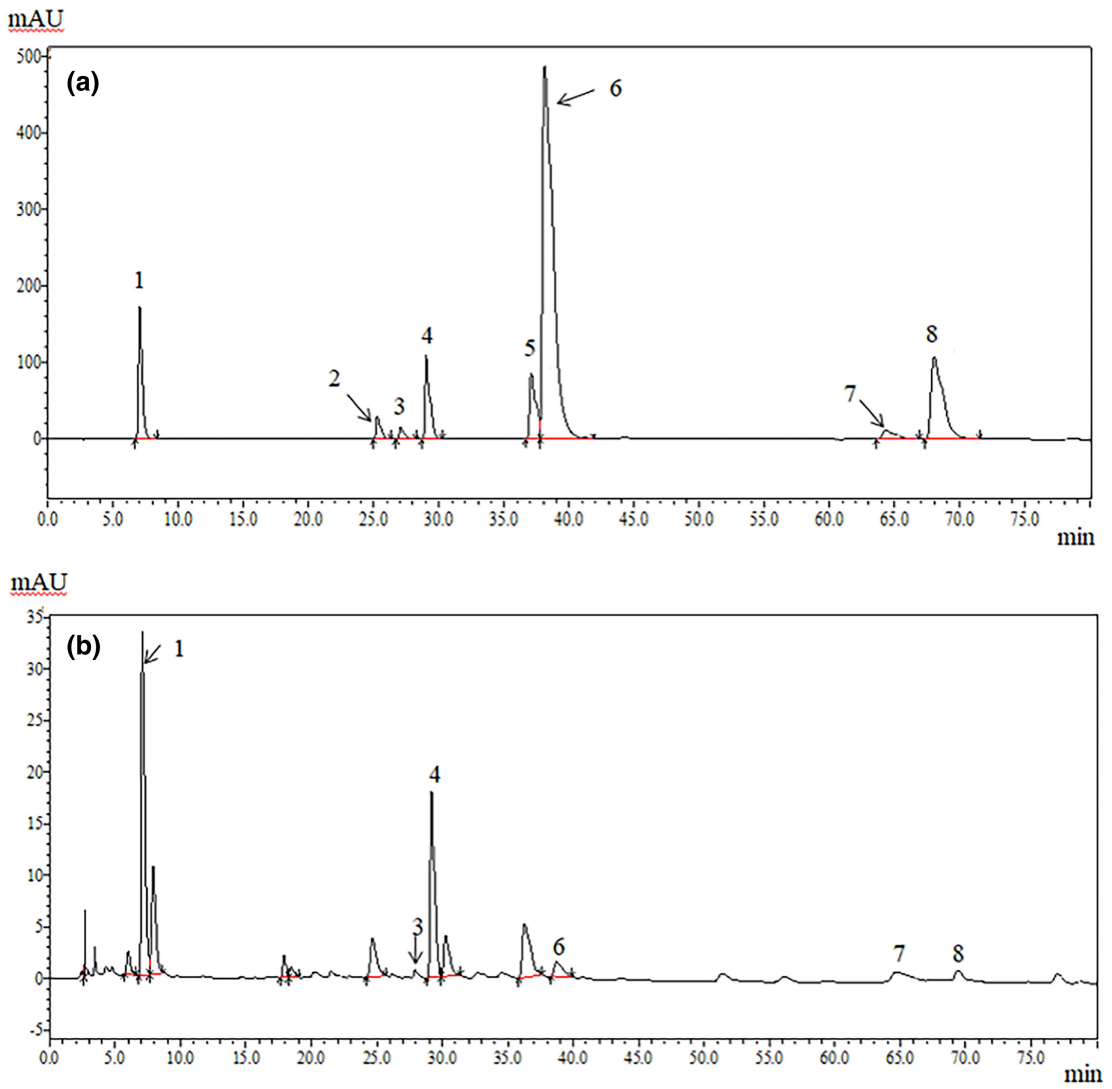
Chromatograms of tea polyphenol mixed standards (a) and tea phenolic extracts of *KBT* (b). The total number of peaks in this figure coincides with that of the detected compounds in Table 2.

According to the standard curve of gallic acid concentration and absorbance, *y* = 0.0024*x* + 0.0323 (*R* ^2^ = 0.9988), where y represents absorbance and x represents gallic acid mass concentration, and the total KBT polyphenol content was 282.19 ± 6.47 mg GAE/g DW, as calculated from the regression equation. HPLC showed that there were four main types of polyphenol monomers (Figure 1 and Table 2). The detected compounds included epigallocatechin gallate (82.94 μg/mg), catechin (28.55 μg/mg), gallic acid (20.45 μg/mg), and epicatechin gallate (9.24 μg/mg).

**TABLE 2:** Qualitative and quantitative analysis of polyphenol monomers in KBT by HPLC.

Serial number	Standard	Retention time (min)	Regression equation	Correlation coefficient	Polyphenol concentration (μg/mg)
1	Gallic acid	7.019	y = 30,556*x*−13,608	0.999	20.45
2	Epigallocatechin	25.257	*y* = 1652.7*x*−2659.5	0.994	ND
3	Catechin	27.050	*y* = 807.18*x*−8145.5	0.988	28.55
4	Caffeine	29.035	*y* = 30,122*x* + 5827.1	0.998	14.15
5	Epicatechin	37.086	*y* = 10,576*x*−14,893	0.995	ND
6	Epigallocatechin gallate	38.121	*y* = 9516.1*x*−725,923	0.970	82.94
7	Epicatechin gallate	64.317	*y* = 4955.1*x*−26,561	0.948	9.24
8	Catechin gallate	68.018	*y* = 11,815*x* + 1532.4	0.978	2.71

Abbreviation: ND, not determined.

### 3.2 Effect of KBT polyphenols on Cyp3a11 expression in the liver

**FIGURE 2:**
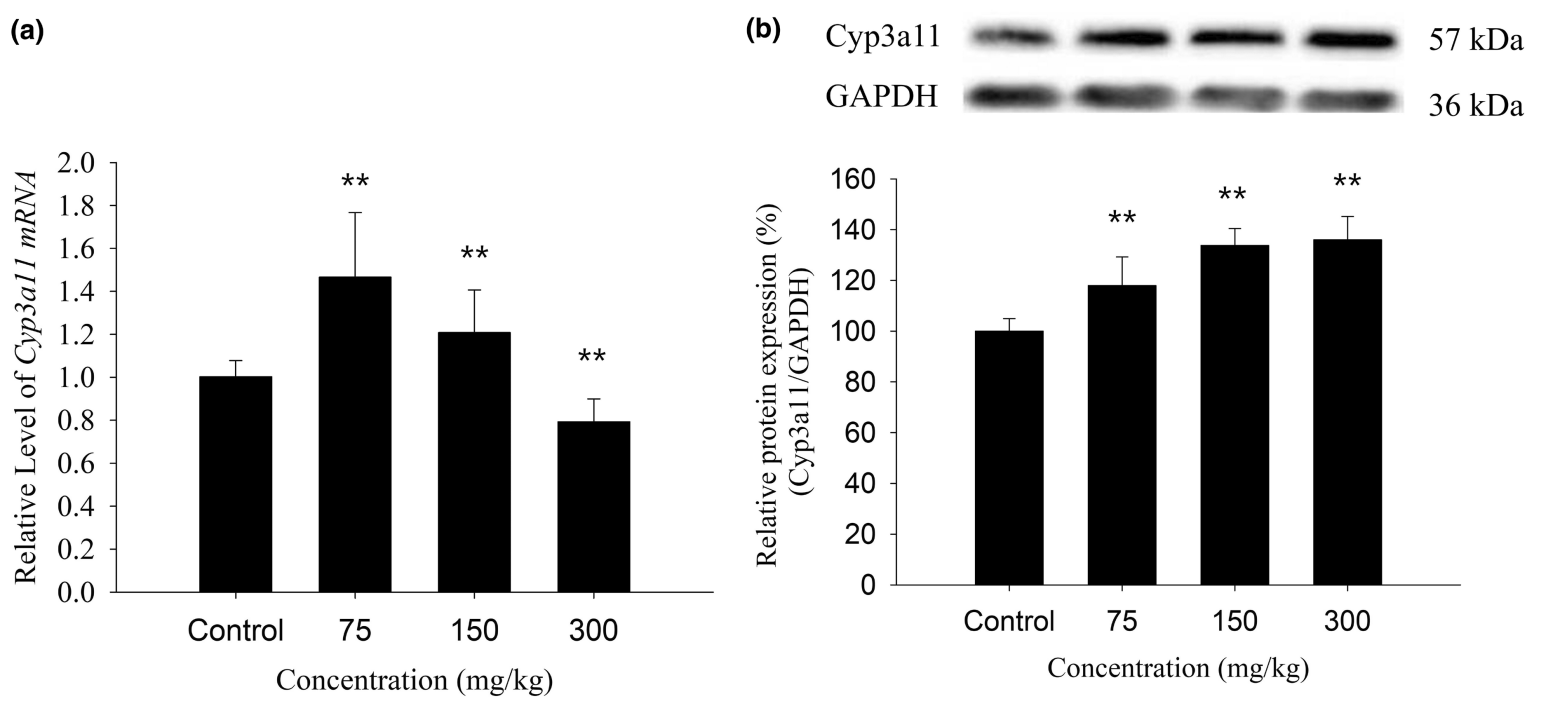
Effects of KBT polyphenolic extracts on the mRNA (a) and protein (b) levels of Cyp3a11 in C57BL/6J mouse liver. The normalized expression data are presented as the mean ± SD (*n* = 6). **p < .01 versus control group.

It was found that a continuous daily administration (7 days) of 75, 150, or 300 mg/kg KBT polyphenols could upregulate Cyp3a11 expression in liver tissues. Among them, 75 mg/kg of KBT polyphenols could increase the Cyp3a11 protein and mRNA levels to 118% and 146%, respectively, when compared with the control mice (*p* < .01). Moreover, 150 mg/kg of KBT polyphenols elevated the Cyp3a11 protein and mRNA levels to 134% and 121%, respecively, compared to the control mice (*p* < .01). Besides, KBT polyphenols at 300 mg/kg reduced the CYP3A11mRNA expression to 79% (*p* < .01) but induced the protein expression to 136% (*p* < .01), when compared to the control mice (Figure 2a,b). Taken together, KBT polyphenols could induce the protein and mRNA levels of the murine Cyp3a11 enzyme.

### 3.3 Effect of KBT polyphenols on Cyp1a2 expression in the liver

**FIGURE 3:**
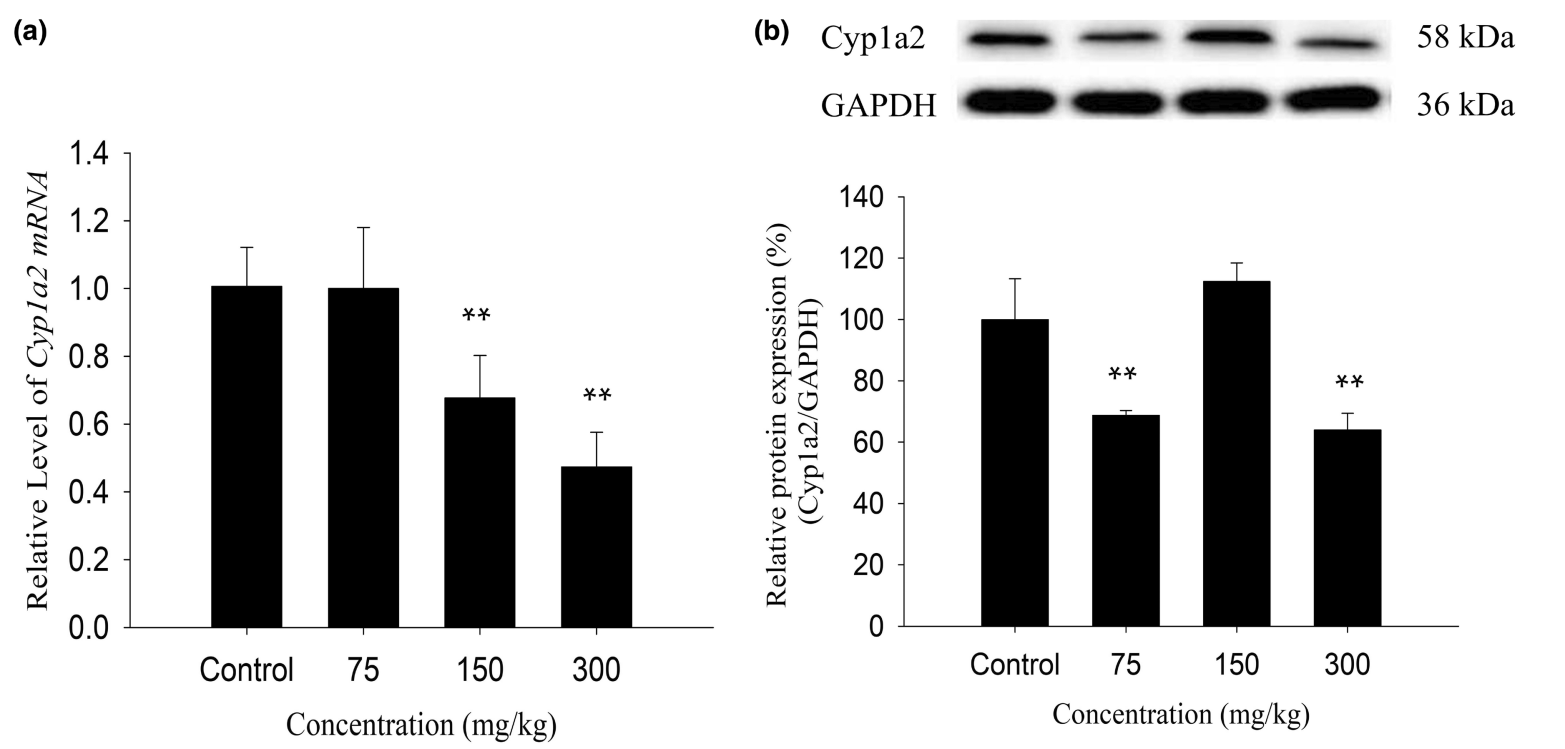
Effects of KBT polyphenolic extracts on the mRNA (a) and protein (b) levels of Cyp1a2 in C57BL/6J mouse liver. The normalized expression data are presented as the mean ± SD (*n* = 6). **p < .01 versus control group.

As shown in Figure 3, 75 mg/kg of KBT polyphenols downregulated the Cyp1a2 protein expression to 69% compared with the control mice (*p* < .01), but not the mRNA expression. When the dose was elevated to 150 mg/kg, the Cyp1a2 mRNA expression levels increased to 67% in comparison with the control group (*p* < .01), but not the protein expression. At the highest dose of 300 mg/kg, the Cyp1a2 protein and mRNA levels were 64% and 47%, respectively, in comparison with the control group (*p* < .01). In conclusion, KBT polyphenols can downregulate the protein and mRNA levels of the mouse Cyp1a2 enzyme.

### 3.4 Effect of KBT polyphenols on Cyp2e1 expression in the liver

**FIGURE 4:**
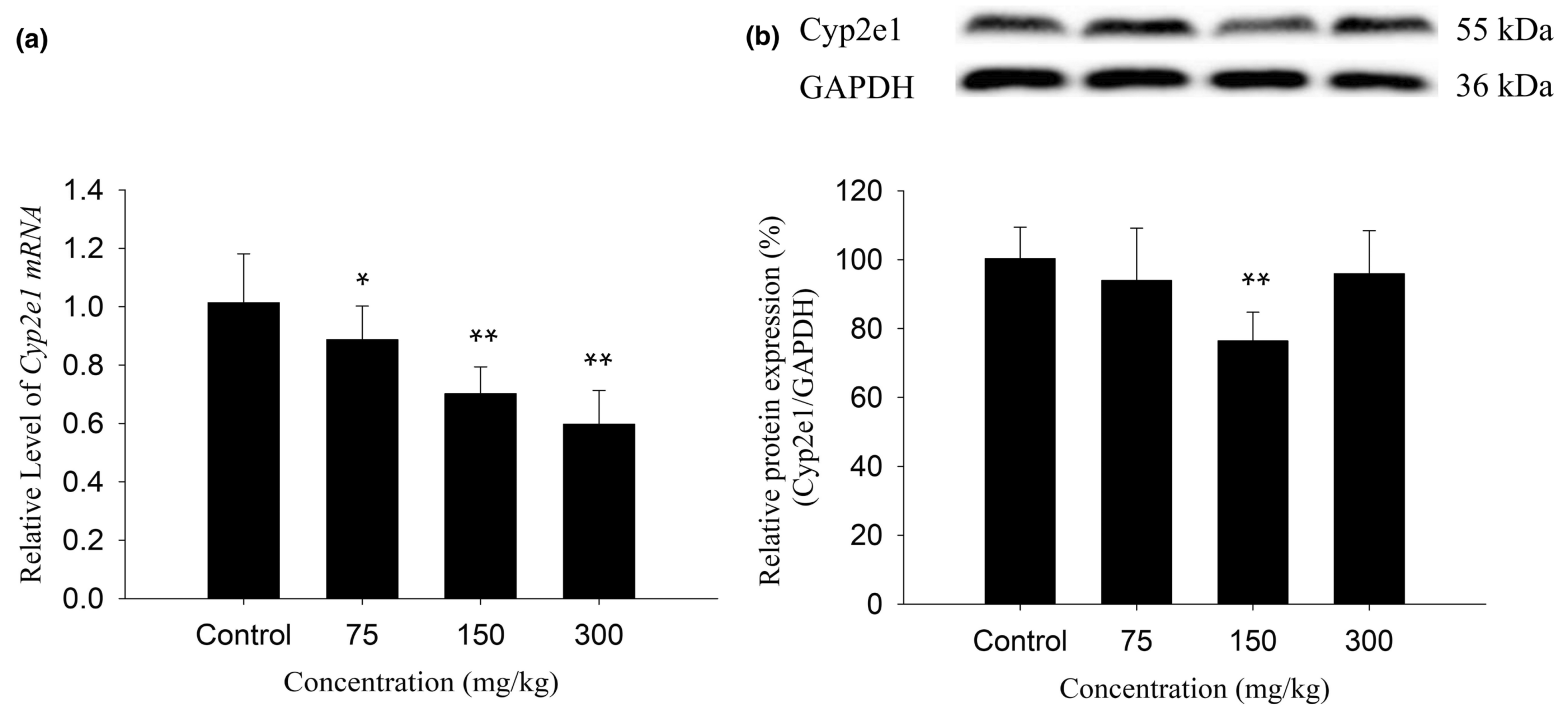
Effects of KBT polyphenolic extracts on the mRNA (a) and protein (b) levels of Cyp2e1 in C57BL/6J mouse liver. The normalized expression data are presented as the mean ± SD (*n* = 6). *p < 0.05 versus control group, **p < .01 versus control group.

As described in Figure 4, compared with the control group, the 75 mg/kg and 300 mg/kg groups showed significant inhibition of mouse hepatic Cyp2e1 mRNA expression, downregulating it to 89% and 60%, respectively (*p* < .05, *p* < .01), but had no significant effect on its protein expression, and the 150 mg/kg group showed significant inhibition of its mRNA and protein expression, downregulating it to 70% and 76%, respectively (*p* < .01, *p* < .01). Overall, Cyp2e1 expression was inhibited.

### 3.5 Effect of KBT polyphenols on Cyp2c37 expression in the liver

**FIGURE 5:**
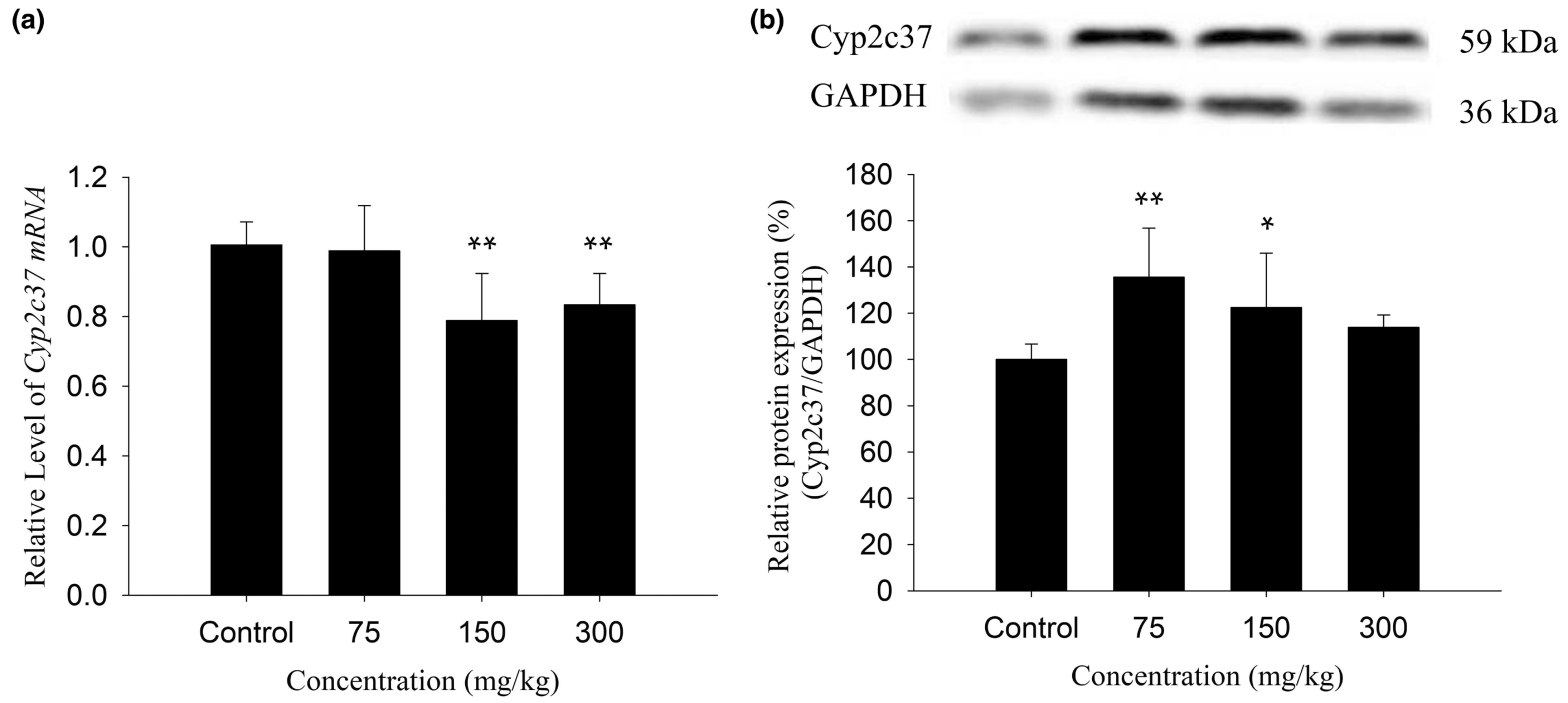
Effects of KBT polyphenolic extracts on the mRNA (a) and protein (b) levels of Cyp2c37 in C57BL/6J mouse liver. The normalized expression data are presented as the mean ± SD (*n* = 6). *p < 0.05 versus control group, **p < .01 versus control group.

As shown in Figure 5, the 75 mg/kg group had no significant effect on mRNA expression but significantly induced protein expression, which increased to 135% (*p* < .01). The 150 mg/kg group significantly inhibited its mRNA expression but significantly induced its protein expression to 79% and 122%, respectively (*p* < .01, *p* < .05). The 300 mg/kg group significantly inhibited mRNA expression but had no significant effect on protein expression, and mRNA expression was downregulated to 83% (*p* < .01).

### 3.6 Effect of KBT polyphenols on PXR expression in mouse liver

**FIGURE 6:**
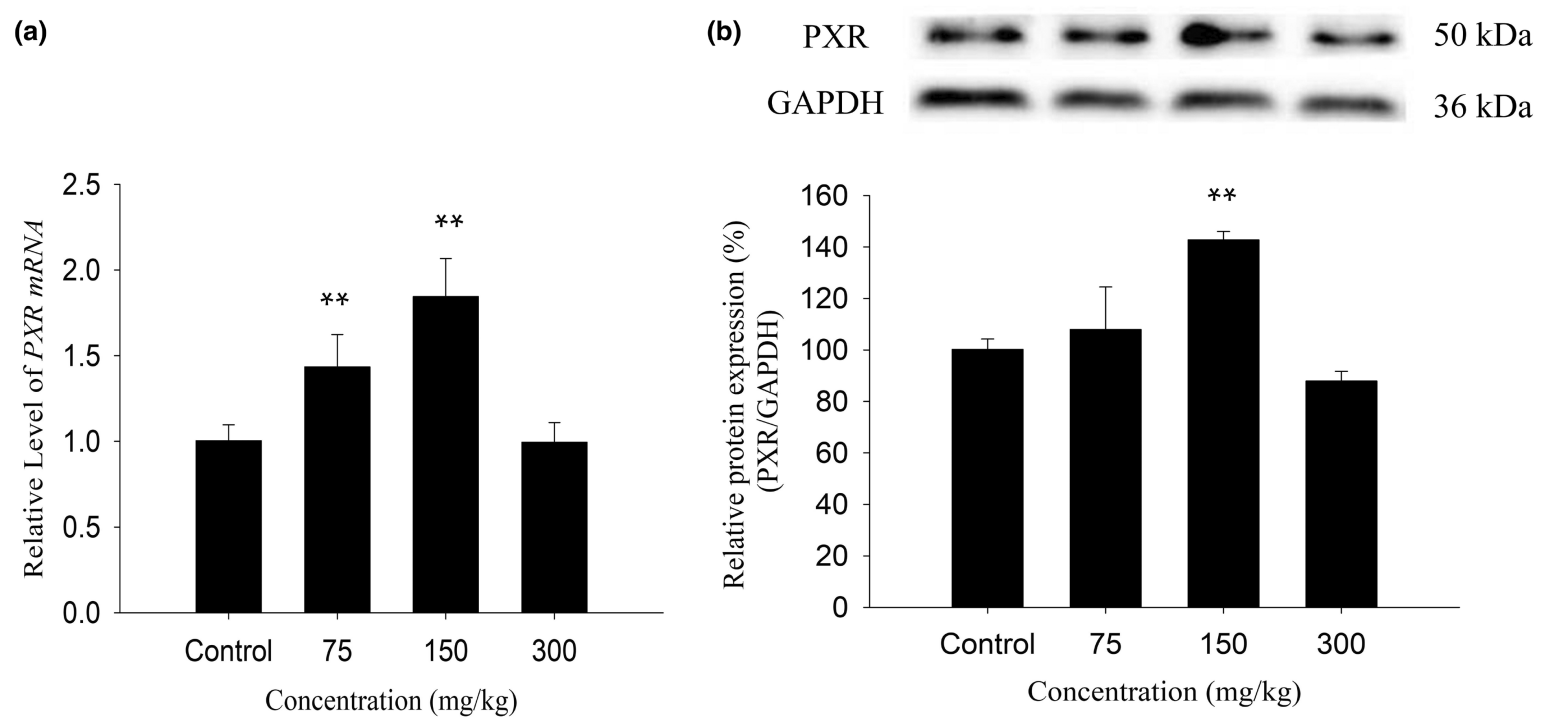
Effects of KBT polyphenolic extracts on the mRNA (a) and protein (b) levels of PXR in C57BL/6J mouse liver. The normalized expression data are expressed as the mean ± SD (*n* = 6). ** *p* < .01 versus control group.

To confirm whether the inductive effect of KBT polyphenols on Cyp3a11 is related to the activation of PXR, the protein and mRNA expression of PXR in the mouse liver were detected. As shown in Figure 6, 75 mg/kg of KBT polyphenols upregulated the PXR mRNA expression by 43% compared with the control mice (*p* < .01), but not the protein expression. When the dose was elevated to 150 mg/kg, the PXR mRNA and protein levels increased by 84% and 43% in comparison with the control group (*p* < .01). At the highest dose of 150 mg/kg, the PXR protein and mRNA levels were not noticeably different compared with the control mice (*p* > .05). Overall, KBT polyphenols, particularly at 150 mg/kg, could increase PXR expression. We speculate that the inductive effect of KBT polyphenols on CYP3A111 may be realized by PXR expression.

### 3.7 Effect of KBT polyphenols on the PXR‐CYP3A4 dual luciferase reporter gene

**FIGURE 7:**
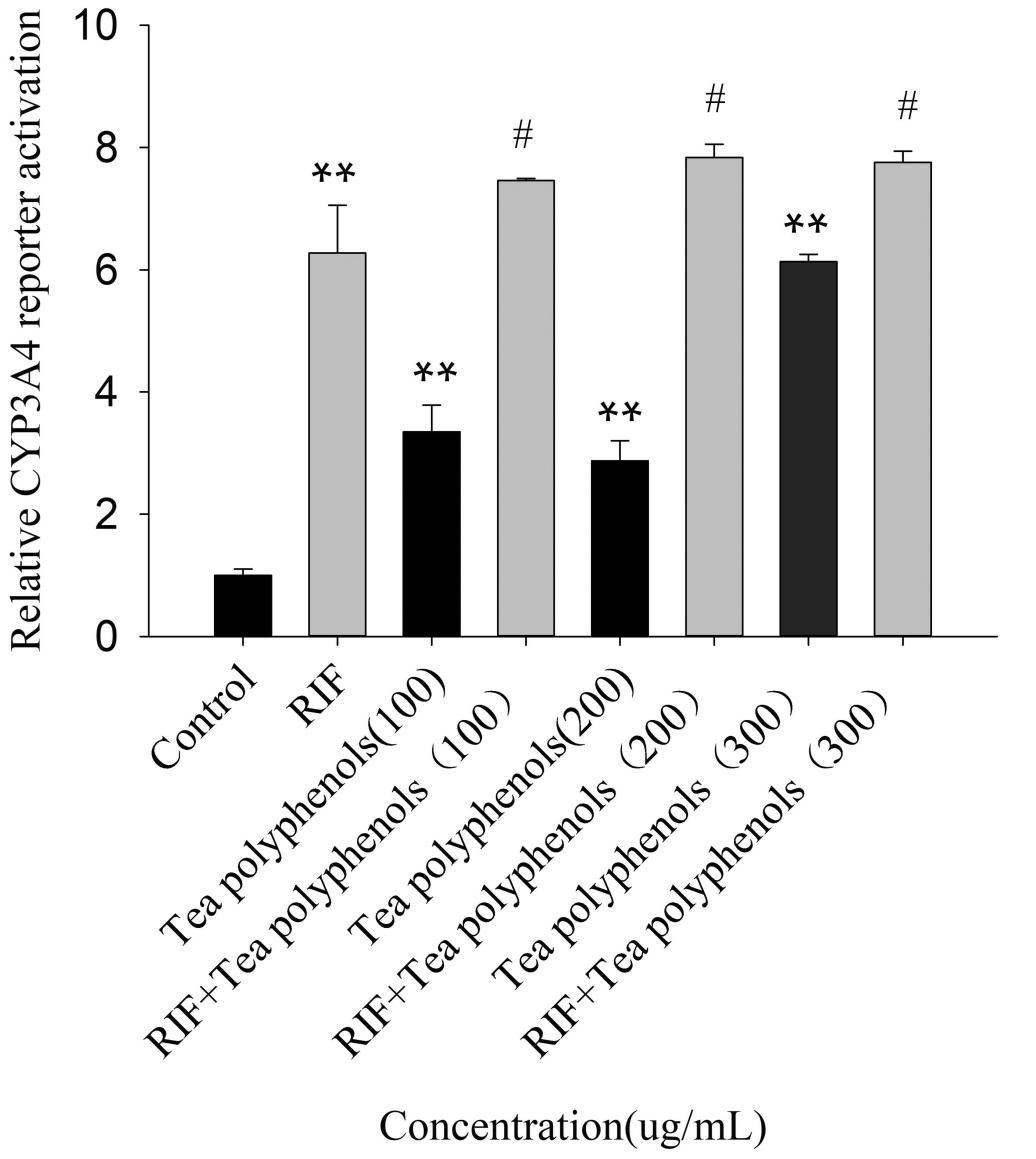
Effect of KBT polyphenolic extracts on the PXR‐mediated transactivation of the CYP3A4 promoter in HepG2 cells transfected with the pGL3‐CYP3A4‐XREM‐Luc reporter gene or hPXR expression vector. The cells were exposed to RIF (10 mM) or DMSO (0.1%, vehicle) in the presence or absence of KBT polyphenolic extract (100, 200, and 300 μg/mL) for 24 h. Luciferase activities were subsequently measured. The data are shown as fold‐change values relative to the hPXR‐transfected cells (control group, *n* = 3). **p < .01 versus solvent control group, ^#^ *p* < .05 versus RIF‐treated group (*n* = 3).

To further affirm the inductive effects of KBT polyphenols on human CYP3A4 mRNA expression, the PXR‐mediated transactivation of the CYP3A4 promoter in HepG2 cells transfected with the pGL3‐CYP3A4‐XREM‐Luc reporter gene or hPXR expression vector was determined by using a dual‐luciferase reporter assay. RIF (10 mM) and DMSO (0.1%) were employed as standard inducers and untreated controls, respectively. As shown in Figure 7, the activity of CYP3A4 luciferase in the RIF treatment group was remarkably higher (6.27 times) than that in the control group (*p* < .01), suggesting that the highly sensitive PXR‐CYP3A4 luciferase reporter gene system has been successfully established. Besides, a significant inductive effect was observed when 100, 200, and 300 μg/mL of KBT polyphenols were treated alone (*p* < .01). Moreover, KBT polyphenols significantly increased RIF‐induced CYP3A4 reporter activity (*p* < .05), indicating that KBT polyphenols and RIF could synergistically induce CYP3A4 activity through the PXR pathway. These results suggest that KBT polyphenols may function as a direct or indirect agonist of PXR.

## 4 DISCUSSION

In this paper, we investigated the quantitative and qualitative effects of KBT polyphenols on four major isoforms of mouse liver Cyp450s, Cyp3a11, Cyp2e1, Cyp2c37, and Cyp1a2, at both the transcriptional level and the protein expression level, and explored their possible mechanisms by nuclear receptor PXR expression and Dual Luciferase Reporter Gene Assay.

Tea polyphenols are naturally occurring substances that are widely consumed for their beneficial effects on human health (Zhang, Qi, & Mine, 2019). However, limited information is available on the roles of tea polyphenols in regulating CYP450. CYP450 is well recognized as the most prominent enzyme responsible for drug/xenobiotic metabolism, and it can be transcriptionally regulated by the PXR nuclear receptor. In this work, we demonstrated for the first time that KBT polyphenols could significantly upregulate CYP3A11/3A4 expression in the liver by activating the PXR‐CYP3A4 pathway.

Previous reports on the effects of tea polyphenols on CYP450 have been relatively inconsistent and even contradictory. Black tea could effectively inhibit drug metabolism by regulating CYP3A4 activities (Radeva‐Llieva et al., 2022). Donovan et al. (2004) also suggested that decaffeinated green tea tended to change the composition of drugs in healthy controls, mainly through the CYP3A4 or CYP2D6 metabolism pathways. Matoušková et al. (2014) studied the in vivo effects of green tea extract on drug‐metabolizing enzymes and observed that high concentrations could affect drug effectiveness and safety. In this experiment, an intermediate dose of KBT polyphenols significantly increased the Cyp3A11 levels in the liver, suggesting that KBT polyphenols can upregulate the activity of drug‐metabolizing enzymes.

Interestingly, we observed that 300 mg/kg of KBT polyphenols decreased Cyp3a11 mRNA expression, but increased Cyp3a11 protein expression. Similar studies have shown that the correlation between RNA and proteins is not always straightforward but favors intrinsic dependence (Payea et al., 2023). Different regulatory mechanisms (e.g., degradation and synthesis rate) have different impacts on protein and mRNA synthesis and ultimately affect the molecular weight (Drees et al., 2023). Furthermore, the overall protein level can be regulated by other post‐transcriptional and/or post‐translational events (Schwanhäusser et al., 2013).

Additionally, we found that KBT polyphenols could upregulate the PXR mRNA and protein level, indicating that the effect mechanism on CYP450 may be through the regulation of PXR. To verify this conjecture, we used the Dual Luciferase Reporter Gene Assay to confirm whether KBT polyphenols are an exogenous ligand of PXR. The reporter gene of PXR is established according to the regulation of CYP3A4 expression by PXR. Therefore, we can understand the induction of CYP3A4 by exogenous active substances, and CYP3A4 activation plays a role through PXR. Our results suggest that KBT polyphenol extract enhances the transcriptional activation of the CYP3A4 promoter via PXR, which triggers the activation of PXR and CYP3A4 in human liver‐like cells, leading to the upregulation of their transcriptional regulation. As a key xenobiotic receptor, the activation of PXR can be mediated by many xenobiotics, including a wide range of therapeutic and herbal medicines (Poudel et al., 2023). In our previous study, we described that ginsenosides could enhance the RIF‐induced PXR transactivation of the CYP3A4 promoter and stabilize CYP3A11/3A4 expression in liver tissue after injury (Sun et al., 2019).

Many studies have demonstrated that drug–drug interaction is the main clinical implication for PXR activation, of which PXR is the key transcriptional regulator of CYP3A4 (Hu et al., 2019; Vyhlídalová et al., 2020). PXR can act as a xenobiotic detector, owing to its wide target binding range, whose activities are mainly affected by different exogenous stimuli, leading to the alteration of its structural characteristics (Zhang et al., 2023). Besides, the PXR‐CYP3A pathway is not a closed reaction mode. The regulatory region of CYP3A4 binds to a variety of nuclear receptors, thereby promoting or inhibiting their gene expression. Constitutive androstane receptor (CAR) and PXR could mediate the transcriptional induction of CYP3A interactively (McMillan et al., 2018; Zhang et al., 2022). In addition, many factors can influence CYP3A4 expression, such as factors affecting the binding of the nuclear receptor to CYP3A4, competitive inhibition caused by direct binding of the substance to the enzyme, or metastatic effects (Wang et al., 2021).

## 5 CONCLUSIONS

In conclusion, we found that KBT polyphenols significantly induced the expression of CYP450 via the PXR‐CYP3A4 pathway. This study will improve the rational use of KBT to maximize the benefits and minimize the risks for those who take the medication. KBT is a popular tea drink, and many people have the habit of drinking tea while taking medication. This dietary pattern may lead to unexpected interactions between tea and drugs. Therefore, more research is needed to fully predict the effects of KBT on the metabolic behavior of drugs or chemicals.

## FUNDING INFORMATION

This research was funded by the Marine Medicine Innovation Platform for the Integration of Production and Education Project of the Guangdong Provincial Education Department (No. 2021CJPT014), the Shenzhen Stability Support Project for Colleges and Universities (No. 20220814205518001), the Shenzhen Sustainable Development Project (KCXFZ20230731094501002) and the Guangdong Education Department (2021KCXTD069).

## CONFLICT OF INTEREST STATEMENT

The authors declare that they have no conflict of interest.

## Data Availability

The data sets used and/or analyzed during the current study are available from the corresponding author on reasonable request.
